# Breaking the metabolo-immune cycle in primary biliary cholangitis for therapeutic benefit

**DOI:** 10.3389/fimmu.2026.1766177

**Published:** 2026-03-25

**Authors:** Yan Song, Hui Wang, Shiyu Du, Yuqing Li, Di Yang, Min Xu, Qinglong Jin

**Affiliations:** 1 Department of Hepatology, Center of Infectious Diseases and Pathogen Biology, The First Hospital of Jilin University, Changchun, China; 2 Jilin Provincial Key Laboratory of Metabolic Liver Diseases, The First Hospital of Jilin University, Jilin University, Changchun, China; 3 China-Singapore Belt and Road Joint Laboratory on Liver Disease Research, The First Hospital of Jilin University, Jilin University, Changchun, China

**Keywords:** bile acids, cholangiocytes, fibrosis, immunity, metabolism, microenvironment

## Abstract

Primary biliary cholangitis (PBC) is a chronic autoimmune liver disease characterized by cholestasis-driven bile duct injury and fibrosis. Biliary epithelial cells (BECs) play a central role in both bile homeostasis and immune regulation, and their metabolic and immune dysfunction is critical in PBC pathogenesis. Furthermore, systemic metabolic disturbances, such as gut dysbiosis, contribute to disease progression. This paper systematically examines the interplay between BEC metabolism—including bile acid imbalance and lipotoxicity—and immune dysregulation, such as autoantigen exposure and Th1/Th17 responses, highlighting how these interactions fuel a self-sustaining “metabolo–immune–fibrosis” cycle in PBC. Current and emerging therapies are also reviewed, emphasizing that future management should move beyond single-pathway targeting and pursue combination strategies capable of simultaneously modulating metabolic, inflammatory, and immune networks.

## Introduction

Primary biliary cholangitis (PBC) is a chronic autoimmune cholestatic liver disorder that predominantly affects women aged 40 to 70 years (1). It has an estimated global prevalence of 1.91 to 39.2 per 100,000 individuals and an annual incidence of 0.23 to 5.31 per 100,000, with significant geographic and ethnic variability (1). Ursodeoxycholic acid (UDCA) is the first-line treatment; however, the response is inadequate in 20–40% of patients (2). Although UDCA can improve biochemical parameters and delay histological progression in responsive cases, it has only limited efficacy in reversing established fibrosis or preventing disease progression at advanced stages. PBC can also be expensive; real-world claims data show that annual acute care costs for PBC patients with cirrhosis and at least one acute event average $113,568, compared to $47,436 for non-cirrhotic patients, demonstrating how the economic burden intensifies with disease progression (3). Therefore, the management of PBC continues to pose considerable challenges, highlighting the need for in-depth research into its pathogenesis to guide the development of more effective therapeutic approaches.

The pathogenesis of PBC is multifactorial, involving a complex interplay of genetic, environmental, metabolic, and immune factors (4–7). Under physiological conditions, biliary epithelial cells (BECs) play a vital role in maintaining metabolic homeostasis through bile secretion and detoxification. In PBC, however, immune-mediated injury transforms BECs into drivers of metabolic dysregulation, characterized by impaired bile acid handling and the initiation of pro-inflammatory signaling. Recent studies have emphasized that metabolic reprogramming of BECs—including aberrant bile acid synthesis and lipid peroxidation—can activate pro-inflammatory pathways (8). Concurrently, chronic inflammation mediated by immune cells further exacerbates metabolic disturbances, creating a self-reinforcing cycle (9). Clinically, therapies targeting metabolic pathways have demonstrated superior efficacy to immunomodulatory strategies, suggesting that metabolic dysregulation may represent a central pathogenic mechanism beyond immune dysfunction. This review proposes that a self-sustaining metabolo–immune positive feedback loop originating in BECs constitutes the core driver of PBC pathogenesis.

## Metabolo-immune dysfunction of BECs: the initiating factor in the pathological cycle of PBC

BECs tightly regulate bile flow, composition, and pH by absorbing bile acids (BAs) and secreting HCO_3_^-^ and water. This process is mediated by apical aquaporin 1 (AQP1), which facilitates water permeation to maintain bile fluidity, and the cystic fibrosis transmembrane conductance regulator (CFTR), which secretes Cl^-^ to drive anion exchanger 2 (AE2)-mediated HCO_3_^-^ export, thereby generating an alkaline “bicarbonate umbrella” that shields BECs from hydrophobic bile acid toxicity (4, 10, 11). These secretory processes are co-regulated by secretin/VIP via the cAMP-PKA pathway and by acetylcholine/ATP via the Ca²^+^-PLC pathway (12, 13). The underlying mechanism involves the activation of the cAMP-PKA pathway, which phosphorylates and opens the CFTR chloride channel. This increases the availability of luminal Cl^-^, thereby driving AE2-mediated HCO_3_^^-^^ secretion and ultimately reinforcing the protective function of the bicarbonate barrier (14, 15). BECs also form a physical barrier through tight junctions and sense mechanical and chemical stimuli such as fluid shear stress, bile acids (BAs), and ATP via primary cilia (16–18). Additionally, they reinforce a chemical barrier via IgA and β-defensin secretion and downregulate the FXR–ASBT axis to limit BA retention during cholestasis (19, 20). In PBC, BEC metabolic dysfunction initiates immune injury. Key pathological steps include:

### Metabolic dysregulation activates immunity

Impaired HCO_3_^-^ secretion—due to AE2 dysfunction or miR-506 overexpression—disrupts the bicarbonate umbrella, leading to bile acidification and accumulation of hydrophobic toxic BAs, which induce BEC apoptosis (21, 22). Apoptotic cells release self-antigens such as PDC-E2. These are taken up by antigen-presenting cells, and molecular mimicry (e.g., with *Escherichia coli* or Epstein–Barr virus antigens) breaks immune tolerance, triggering anti-mitochondrial antibody (AMA) production and autoimmunity (23–25). Notably, under the stimulation of IFN-γ, BECs upregulate MHC class II molecules and CD40, acquiring the function of non-professional antigen-presenting cells (21, 26). This enables BECs to directly present self-antigens to CD4^+^ T cells, driving the extension of immune attack from the initial target PDC-E2 to other self-components (e.g., sp100, gp210) (27). This BEC-mediated antigen presentation allows immune injury to persist even after the initial triggers have subsided, thereby sustaining the pathological process.

### Immune attack amplifies metabolic defects

Activated CD4^+^ and CD8^+^ T cells—particularly CD103^+^ tissue-resident memory T cells—directly kill PDC-E2–expressing BECs via granzyme and perforin release (24). Th1- and Th17-derived cytokines such as IFN-γ and IL-17 exacerbate inflammation (28, 29). B cells produce antibodies that form immune complexes, activate complement, and present antigen, thereby sustaining T-cell activation and amplifying injury (30–32). Macrophages, especially the M1 subset, further drive T-cell responses and fibrosis via IL-12, TNF-α, and antigen presentation (33). Beyond effector cell attack, functional defects in regulatory T cells (Tregs) are critical for the failure of immune tolerance. The frequency and suppressive function of Tregs are significantly reduced in patients with PBC, and BA metabolites (e.g., isoalloLCA) can directly regulate the Treg/Th17 balance via the RORγt/FOXP3 axis, promoting effector T cell polarization (34). This Treg/Th17 imbalance, particularly alterations in the tissue-resident capacity of Tregs, further disrupts immune homeostasis in the biliary microenvironment and accelerates disease progression (35).

### Self-sustaining metabolo–immune cycle

Metabolic disturbances in PBC—such as lipid dysregulation and mitochondrial dysfunction due to reduced PPAR-α activity—increase reactive oxygen species (ROS) and accelerate apoptosis (36, 37). Single-cell studies have identified a DUOX2^+^ACE2^+^ subset of small cholangiocytes enriched in PBC livers (38, 39). Based on its high expression of the ROS-generating enzyme DUOX2, this subset is proposed to induce local oxidative stress and impair bile secretion, potentially worsening cholestasis. These cells colocalize with portal immune infiltrates, suggesting a role in metabolo–immune crosstalk. However, direct experimental evidence establishing their functional contribution to PBC pathogenesis is lacking.

In summary, BEC metabolo–immune failure initiates a local vicious cycle: bicarbonate umbrella disruption → toxic bile acid retention → autoantigen exposure → AMA/T-cell–mediated attack. This establishes a tipping point for subsequent intrahepatic and systemic escalation of disease.

## Metabolo-immune imbalance drives a vicious cycle and systemic amplification in the microenvironment

In the pathogenesis of PBC, metabolic dysfunction and immune-mediated injury of BECs are not parallel processes; rather, they are coupled through the central hub of cellular senescence, forming a self-reinforcing vicious cycle. Senescent cells act as “signal relays” and “inflammatory amplifiers”, converting transient local damage into persistent pro-inflammatory signals with systemic consequences.

Cholestasis-induced accumulation of toxic BAs and oxidative stress are primary drivers of BEC senescence (40). Concurrently, cytokines such as IFN-γ and TNF-α released by activated T cells (particularly CD8^+^ tissue-resident memory T cells) and macrophages (41, 42) synergize with metabolic stressors to promote BEC entry into senescence. BECs in liver biopsies from PBC patients overexpress senescence markers, including p16, p21, and senescence-associated β-galactosidase (SA-β-Gal) (43, 44). Spatial transcriptomic analysis has further revealed that senescent bile ducts are surrounded by immune and stromal cells expressing SASP-related genes (e.g., *IL6*, *CXCL10*, *TGFB1*), confirming the role of senescent cholangiocytes as hubs driving local inflammation (44).

In the early amplification phase of this vicious cycle, liver-enriched innate-like lymphocytes are the first to respond to metabolic stress, bridging innate and adaptive immunity. Mucosal-associated invariant T (MAIT) cells secrete IL-17 into the cholestatic microenvironment, directly promoting bile duct injury (45). γδ T cells sense stressed BECs via NKG2D receptors and rapidly release IFN-γ and granzymes, linking innate and adaptive immunity (46). Innate lymphoid cells (especially ILC1 and ILC3) are aberrantly activated in the cholestatic microenvironment, exacerbating inflammation and driving fibrosis through IFN-γ and IL-17 secretion (47). These innate effectors cooperate with metabolic stressors to establish a pro-inflammatory niche that favors the induction of cellular senescence.

The senescence-associated secretory phenotype (SASP) refers to the complex repertoire of factors secreted by senescent cells, including pro-inflammatory cytokines (IL-6, IL-8), chemokines (CXCL10, CCL2), growth factors (TGF-β, HGF), and matrix-remodeling enzymes (MMPs) (48). Unlike apoptosis or necrosis, senescent cells do not die immediately but function as activated secretory cells, amplifying local stress signals into systemic inflammatory signals via the SASP. It is important to emphasize that the SASP is not static but represents a plastic phenotype shaped by cell type and microenvironmental cues. In PBC, several SASP factors have been directly implicated: CXCL10 levels are significantly increased in liver tissue and serum from patients, primarily secreted by senescent BECs and surrounding macrophages, recruiting CXCR3^+^ T cells to bile ducts and sustaining local immune activity (49, 50). TGF-β activates hepatic stellate cells, promoting extracellular matrix deposition and periductal fibrosis; notably, TGF-β exhibits context-dependent duality; while it functions as an anti-inflammatory cytokine in immune homeostasis (51), within the SASP milieu it adopts a pro-fibrotic role (52), highlighting its complex contribution to the PBC microenvironment. IL-6 and TNF-α exacerbate metabolic dysfunction by impairing mitochondrial function, suppressing expression of key enzymes involved in BA metabolism and downregulating transporters, thereby creating a microenvironment conducive to lipid accumulation and further metabolic dysregulation (53). Oxidative stress amplifies BA-triggered immune responses by activating key pro-inflammatory pathways such as NF-κB and JAK-STAT (54, 55).

Of note, within the complex PBC immune microenvironment, immune cells themselves may undergo either senescence or exhaustion, two states with distinct biological implications. Exhaustion, primarily observed in T cells, is a state of hypofunctionality induced by chronic antigenic stimulation and characterized by sustained high expression of inhibitory receptors (e.g., PD-1, TIM-3) and reduced production of effector cytokines, while retaining partial proliferative capacity (56). In contrast, senescence involves stable cell cycle arrest and SASP release, potentially affecting multiple immune lineages (57). In PBC, CD8^+^ T cells predominantly exhibit an exhausted phenotype: single-cell studies demonstrate that liver CD8^+^ tissue-resident memory T cells express high levels of PD-1 and TIM-3 while retaining cytotoxic molecules, a profile consistent with chronic autoimmune stimulation. Notably, PD-1-targeted CAR-T cells can selectively eliminate these cells and ameliorate autoimmune cholangitis in experimental models (58). Senescent states and SASP mechanisms in macrophages and subsets of CD4^+^ T cells in PBC require further validation. T cell exhaustion can be partially reversed by immune checkpoint blockade, albeit with the risk of exacerbating autoimmunity (59); senescent cells, conversely, require targeted elimination using senolytics or senomorphics (60). Distinguishing these states is therefore critical for precision intervention. Future studies should delineate the exhaustion/senescence profiles of distinct immune subsets in PBC and their contributions to disease progression. At the circulating biomarker level, correlations between serum SASP factors (e.g., IL-6, CXCL10) and response to UDCA therapy warrant investigation. At the tissue level, multiplex immunofluorescence should be used to validate associations between senescent bile duct density and inflammatory activity or fibrosis stage. Such efforts could provide a rationale for incorporating SASP biomarker monitoring into clinical trials of UDCA or obeticholic acid (OCA) and for exploring senolytic therapies in PBC.

**Figure 1:**
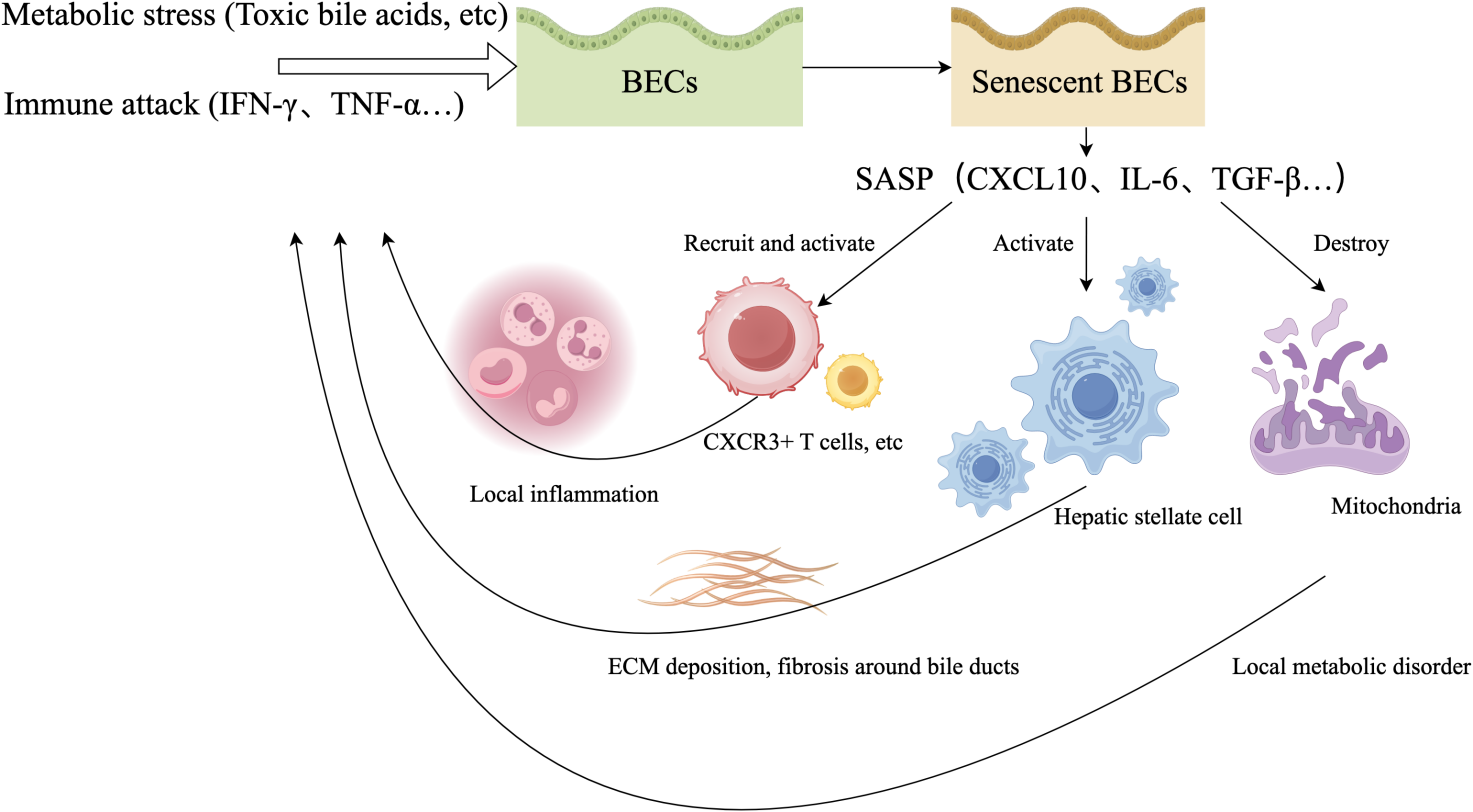
Senescence and SASP-mediated vicious cycle in BECs under metabo-immune stress in PBC. In PBC, BECs undergo senescence triggered by metabolic stress (e.g., toxic bile acids, lipotoxicity) and immune assault (e.g., IFN−γ, TNF−α). Senescent BECs secrete the senescence-associated secretory phenotype (SASP)—including chemokines (e.g., CXCL10), cytokines (e.g., IL−6), and pro-fibrotic factors (e.g., TGF−β)—that perpetuates disease progression through three key mechanisms: ① Sustained recruitment and activation of immune cells (e.g., CXCR3^+^ T cells), amplifying local inflammation; ② Activation of hepatic stellate cells (HSCs), leading to ECM deposition and periductal fibrosis; ③ Mitochondrial dysfunction in neighboring cells, worsening metabolic disturbance. Thus, the SASP converts transient injury into a self-sustaining pathological cycle, driving PBC progression.

In summary, the biliary microenvironment in PBC constitutes a spatiotemporally specific pathological ecosystem centered on BEC senescence. Senescence serves not only as a common endpoint of metabolic stress and immune attack but also, via the SASP, as an amplifier perpetuating the vicious cycle. Disrupting this cycle will likely require combinatorial intervention strategies targeting key metabolo-immune nodes: inhibiting initial metabolic stress, blocking senescence-inducing signals, or selectively eliminating senescent cells to interrupt SASP-driven inflammatory self-perpetuation (**Figure 1**).

## Systemic metabolo-immune dysregulation: from the gut to the whole body

PBC pathophysiology extends beyond the liver, forming a systemic network of metabolo-immune dysregulation that originates in the gut, centers on the liver, and involves multiple organs in a temporal cascade of “gut initiation–hepatic amplification–systemic dissemination”. However, the association between the gut microbiota and PBC remains incompletely defined, as current evidence largely consists of cohort-dependent observations influenced by geography, medication, and disease stage.

Several Asian cohort studies have demonstrated gut microbiota dysbiosis in PBC, characterized by reduced short-chain fatty acid (SCFA)-producing bacteria (e.g., *Faecalibacterium, Sutterella*) and expansion of potentially pathogenic genera (e.g., *Veillonella, Streptococcus*) (61–63). The functional relevance of this dysbiosis is emphasized by the finding that fecal butyrate levels are reduced in UDCA non-responders and correlate with impaired MDSC-mediated immune regulation (64). Butyrate promotes MDSC immunosuppressive activity through PPARδ-driven metabolic reprogramming, and its administration alleviates cholangitis in preclinical models (65). However, these findings are markedly cohort dependent and influenced by UDCA use and cirrhosis. Tang et al. showed that UDCA treatment itself partially restores microbiota composition, and baseline microbial features (e.g., *Clostridia* abundance) associate with treatment response (61). Furukawa et al. further reported that reduced *Faecalibacterium* abundance was associated with non-response to treatment, suggesting this genus may have potential value in predicting therapeutic response (62). Thus, specific microbial changes should be understood as pattern-based trends requiring validation in treatment-naïve, multicenter cohorts.

Reduced beneficial bacteria (e.g., *Helicobacter, Faecalibacterium*) impair secondary bile acid metabolism, while specific microbiota-derived bile acids (e.g., 3-oxo-Δ4,6-LCA) directly activate CD8^+^ T cells via androgen receptor antagonism, linking gut microbial metabolism to biliary autoimmunity (55, 66). It should be noted, however, that this mechanism is derive largely from tumor models. Whether AR-antagonizing BAs achieve effective concentrations in the portal circulation of PBC patients and exert direct effects on intrahepatic CD8^+^ T cells requires further investigation.

Key methodological challenges persist. Existing studies are predominantly cross-sectional, so are unable to distinguish causation from consequence. Cholestasis itself alters the intestinal microenvironment, potentially driving secondary microbial changes. Furthermore, microbial profiles vary significantly across geographic regions and medication backgrounds, with no unified core signature identified. Future research should prioritize: (1) large-scale, treatment-naïve prospective cohorts; (2) multi-omics integration (metagenomics, metabolomics, host genetics); and (3) interventional studies (probiotics, fecal microbiota transplantation) to establish causality.

When intestinal barrier function is impaired, bacterial lipopolysaccharide and microbiota-derived metabolites translocate into the portal circulation, triggering hepatic metabolo-immune crosstalk (67). Dysregulated ASBT/OSTα–β transport leads to portal BA accumulation, impairing FXR/TGR5 signaling. Chronic BA exposure may induce TGR5 desensitization, contributing to metabolic shifts from hypermetabolism to energy plateau, which are potentially responsible for clinical features such as cachexia and insulin resistance (68, 69). Serum C4, a BA synthesis marker, may help monitor FXR/TGR5 dysfunction and guide therapy (70). Accumulated BAs directly regulate intrahepatic immune cells through FXR and TGR5 signaling. FXR activation suppresses macrophage NF-κB signaling, while TGR5 signaling blocks inflammasome activation in Kupffer cells (71, 72). However, chronic BA exposure desensitizes these receptors, abrogating their anti-inflammatory effects and establishing a positive feedback loop of metabolic disorder–immune activation.

BAs also modulate systemic processes via FXR/TGR5 signaling, including lipolysis, adipose browning, and appetite regulation (73, 74). In adipose tissue, FXR/TGR5-regulated lipolysis and browning programs are impaired. TGR5 activation promotes white adipose tissue browning and energy expenditure via the cAMP-PKA pathway (74); FXR agonists can also stimulate browning and thermogenesis (75). Vitamin D receptor (VDR) downregulation represents another key node in PBC pathogenesis, releasing inhibition of CYP7A1 and amplifying BA synthesis (76, 77). VDR agonists (e.g., calcitriol) may thus offer dual benefits by modulating BA homeostasis and improving insulin sensitivity.

**Figure 2:**
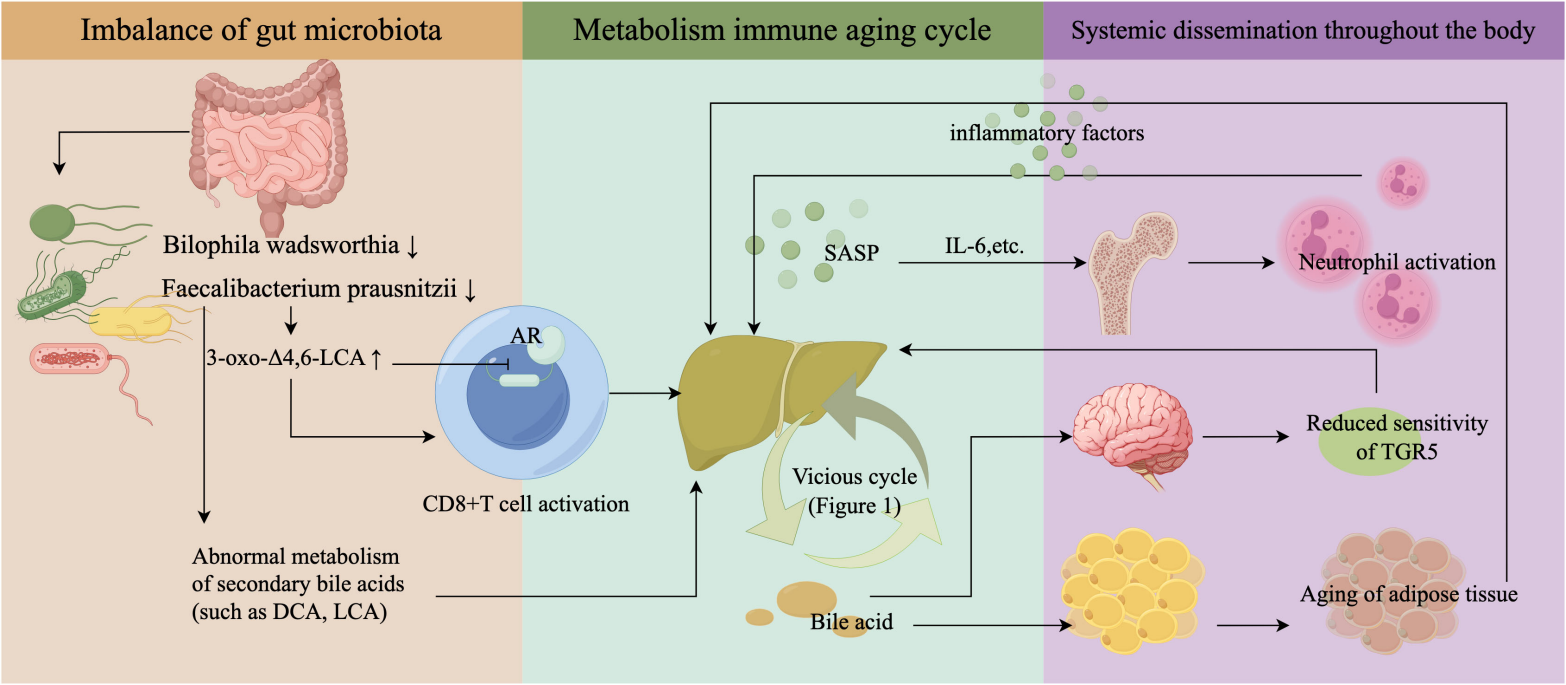
From gut dysbiosis to systemic metabolo-immune dysregulation in PBC. The systemic pathology of PBC originates in the gut and progresses through three key stages: ① Gut Initiation: Dysbiosis reduces beneficial bacteria (e.g., *Helicobacter, Faecalibacterium*), impairing secondary BA metabolism and generating immunomodulatory metabolites like 3-oxo-Δ4,6-LCA. These metabolites activate CD8^+^ T cells via mechanisms such as androgen receptor antagonism, directly linking gut microbiota to hepatic autoimmunity. ② Hepatic Amplification: The local metabo-immune-senescence cycle in the liver (as detailed in **Figure 1**) serves as the core disease amplifier. ③ Systemic Dissemination: Liver-derived SASP factors (e.g., IL-6) and circulating BAs propagate injury systemically: IL-6 pre-activates bone marrow neutrophils; BA overflow desensitizes hypothalamic TGR5, disrupting the “brain-bile axis”; and excessive lipolysis potentially induces adipose senescence, creating a reciprocal “liver-adipose axis” that fuels ongoing inflammation and metabolic dysfunction.

In conclusion, PBC represents a systemic metabolo-immune network disease. While the gut origin hypothesis is intriguing, rigorous causal validation remains essential. Future studies should integrate metagenomics, targeted BA profiling, and multi-organ functional assessment, with strict control of key confounding factors, to elucidate the causal direction of the gut-liver-immune axis and identify clinical intervention targets (**Figure 2**).

## PBC: a paradigmatic metabolo-immune disease and its vicious cycle

As noted above, PBC is not merely an autoimmune disease with metabolic features, but a quintessential “metabolo-immune” disorder. Its pathogenesis is driven by a self-amplifying cycle centered on BECs, where metabolic injury, immune assault, and structural fibrosis continuously reinforce one another, forming the core engine of disease progression.

### Cycle initiation: metabolic damage as a primary immune trigger factor

The cycle begins when metabolic dysfunction in BECs breaches immune tolerance. Hydrophobic BAs (e.g., CDCA) and lipotoxic substances (e.g., oxLDL) act as dual triggers: they induce mitochondrial stress and ROS production, leading to DAMP release and NLRP3 inflammasome activation in macrophages (25); they also promote TNF-α and IL-6 secretion via FXR signaling, recruiting CD27^+^ memory B cells and plasma cells that target BECs (39, 71). Additionally, microbiota-derived BAs like isoDCA inhibit Treg differentiation and promote Th17 responses, further skewing immunity toward autoimmunity (34, 78). Thus, metabolic injury actively converts biochemical stress into specific immune responses.

### Circulation amplification: immune effectors exacerbate metabolic crisis

Activated immune cells directly intensify metabolic dysfunction. CD8^+^ tissue-resident memory T cells and Th1 cells kill BECs, impairing BA homeostasis (58). Inflammatory cytokines (e.g., IFN-γ, TNF-α) disrupt bicarbonate secretion and oxidative stress management in surviving BECs (79). In addition to the classical IFN-γ/IL-17 axis, the IL-12/IL-23/IL-21 pathway is also critical. Intrahepatic macrophages in PBC patients produce IL-23, whose p19 subunit expression correlates with disease stage, and serum IL-23 levels are positively associated with gamma-glutamyl transferase (32). Furthermore, IL-12/Th1 and IL-23/Th17 effector molecules are specifically enriched in the portal tract surrounding bile ducts (80). IL-21 can enhance CD8^+^ T cell cytotoxicity (81), while its role in assisting B cells to produce AMA in PBC still needs to be validated. This immune-mediated metabolic disruption sustains the cycle even as initial triggers fade. A key outcome is BEC senescence, with SASP factors (e.g., IL-6, CXCL10, TGF-β) further recruiting immune cells, activating hepatic stellate cells and disrupting neighboring cell function (82, 83).

### Cycle persistence: structural fibrosis and systemic involvement

Fibrosis acts as a mechanical accelerator. TGF-β from M2 macrophages and senescent BECs activates hepatic stellate cells, causing periductal ECM deposition (82). The resulting architectural distortion worsens cholestasis, reigniting toxic BA accumulation and intensifying the cycle.

**Figure 3:**
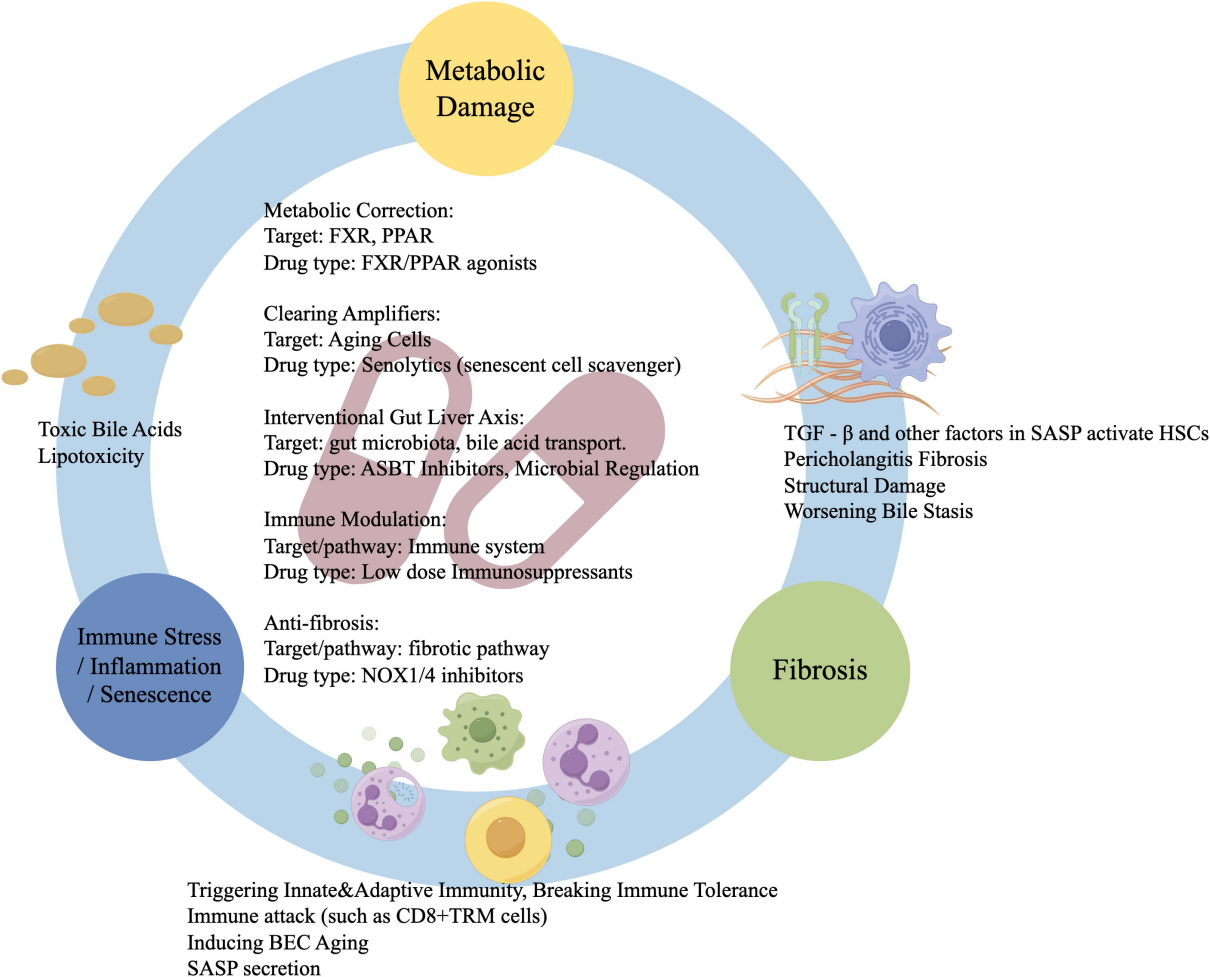
Core vicious cycle and targeted therapies in PBC as a metabolo-immune disease. The self-reinforcing “Metabolism–Immunity–Fibrosis” cycle in PBC progresses through three core stages: Initiation: Metabolic injury in BECs (toxic BAs, lipotoxicity) triggers innate and adaptive immunity, breaking immune tolerance. Amplification: Immune attack (e.g., CD8^+^ TRM cells) and metabolic stress induce BEC senescence. Senescent cells secrete SASP factors, amplifying inflammation and promoting fibrosis. Persistence: SASP components (e.g., TGF−β) activate HSCs, causing periductal fibrosis. Structural distortion worsens cholestasis, reigniting metabolic injury. Therapeutic strategies require multi-node targeting: metabolic correction (FXR/PPAR agonists); immune modulation (low-dose immunosuppressants); amplifier clearance (Senolytics); anti-fibrotic agents (e.g., NOX1/4 inhibitors); gut–liver axis intervention (ASBT inhibitors, microbiota modulation).

The “Metabolo–Immune–Fibrosis” model reframes PBC as a disease of inseparable metabolic and immune dysregulation. This explains why immunosuppression alone fails and UDCA offers only partial efficacy. Future therapies should simultaneously target multiple cycle nodes, e.g., combining FXR agonists to correct metabolism, senolytics to clear inflammatory amplifiers, and antifibrotics to break structural constraints. This perspective not only clarifies pathogenesis but also charts a path toward transformative combination therapy (**Figure 3**).

## Evidence supporting the directionality of cycles: metabolic priority or coevolution?

Defining PBC as a metabolo-immune disease raises a fundamental question: what initiates the vicious cycle? We favor metabolic dysfunction as the initial trigger for immune tolerance breakdown (84), but alternative views persist. The classic “autoimmune-first” theory posits that environmental triggers disrupt self-tolerance, with cholestasis as a secondary event (85, 86), while the “co-evolution” model proposes that metabolic and immune disorders arise in parallel and mutually amplify (87). This section will examine and objectively assess the evidence supporting the “metabolism-first” directionality.

In animal models, simple AE2 dysfunction can lead to BA retention and bile duct cell apoptosis (88), release autoantigens such as PDC-E2, and trigger AMA production, suggesting that metabolic disorders are sufficient to initiate the autoimmune cascade (89). Umemura et al. found using humanized mice that alterations in the BA profile induced by a high-fat diet could significantly amplify cholangitis after immunization and promote its progression (90), indicating that metabolic load is not only a bearer of immune attack but also an active regulator of disease severity. However, these models cannot establish temporal precedence in human disease.

Genetic studies have identified PBC susceptibility loci involved in metabolic regulation (e.g., *VDR, SLC4A2*) (91, 92), but genetic association cannot distinguish cause from consequence, and most genes participate in both metabolic and immune pathways.

Clinical interventions, however, provide important clues. UDCA improves outcomes by correcting BA composition, yet incomplete response rates indicate that metabolic modulation alone is insufficient (1). FXR agonists (i.e., OCA) further improve biochemical indicators over UDCA, but results from the COBALT trial failed to demonstrate an improvement in the primary endpoint, suggesting that biochemical improvement is not equivalent to disease reversal (93). PPAR agonists (elafibranor, seladelpar) both have metabolic regulatory and anti-inflammatory effects, and recent clinical trials have demonstrated significant efficacy (94). By contrast, pure immunomodulators (rituximab, ustekinumab) have demonstrated only limited benefit (95), suggesting that immunosuppression cannot overcome persistent metabolic drivers. Interpretation is complicated, however, by the near-universal use of UDCA as background therapy.

Taken together, the current evidence supports metabolic involvement in PBC pathogenesis but does not definitively establish that metabolic dysfunction precedes immune activation. A more accurate conceptualization may be that metabolic and immune dysregulation engage in bidirectional, self-amplifying co-evolution. Once the cycle is established, origin matters less than identification of intervenable nodes, consistent with core thesis of a metabolo-immune-fibrotic vicious cycle.

## Therapeutic strategies: overcoming the limits of monotherapy by targeting the metabo-immune cycle

The “metabo−immune” model of PBC clarifies why single−pathway therapies show limited efficacy, as they address only isolated components of a tightly interconnected pathological network.

### The limits of monotherapy

The limitations of current therapeutics arise from their incomplete coverage of the vicious cycle. UDCA, the first−line treatment, modestly improves biochemical and histological parameters in responsive patients but fails to reverse fibrosis or prevent progression in 30–40% of cases, reflecting its limited effect on immune dysregulation and the self−sustaining disease cycle (96, 97). Second−line metabolic agents—FXR agonists (e.g., OCA) and PPAR agonists (e.g., elafibranor, seladelpar)—target nuclear receptors to improve cholestasis, but their efficacy is also variable and incomplete against immune and fibrotic components (98–100). OCA’s failure in the COBALT trial to improve hard clinical endpoints highlights the difficulty in modifying established disease (93). Similarly, purely immunomodulatory agents (e.g., rituximab, ustekinumab) have shown limited benefit, as suppressing immunity alone fails to counteract persistent metabolic drivers (101, 102).

### Rationale for combination therapy

Targeting multiple nodes of the metabo−immune cycle simultaneously represents a rational therapeutic advance. A foundational approach builds on UDCA, augmenting it with agents targeting distinct pathological pillars (**Table 1**):

**Table 1:** Clinical trials of combination therapies for primary biliary cholangitis (PBC).

Combination regimen	Core agents	Representative studies (NCT number, phase)	Study characteristics	Key inclusion criteria	Primary endpoints	Current status or available results
Fibrate + UDCA	Fenofibrate/Bezafibrate + UDCA	NCT05751967 (Phase III) NCT06174402 (Phase II/III) NCT06365424 (Phase II/III) NCT06755541 (Phase III)	Focus on UDCA non-responders/inadequate responders	NCT05751967: PBC per criteria^i^; UDCA inadequate responder (Xi’an: ALP >2.5×ULN, AST >2×ULN, or TBIL >1×ULN) after 4-6w UDCA; Age 18-75; BMI 17-28 NCT06174402: PBC per criteria; UDCA inadequate responder (1×ULN < ALP ≤ 1.67×ULN); On UDCA ≥6mo (stable ≥3mo); Age 18-75; BMI 17-28 NCT06365424: Completed prior PBC fenofibrate study (NCT02823353); No prior AE leading to fenofibrate discontinuation NCT06755541: Completed prior PBC fenofibrate study (NCT05751967); No prior AE leading to study drug discontinuation	NCT05751967: ALP + TBIL normalization NCT06174402: Biochemical response (ALP normalization) at 48w NCT06365424: TEAEs NCT06755541: TEAEs	NCT05751967: Recruiting NCT06174402: Active, not recruiting NCT06365424: Recruiting NCT06755541: Recruiting
FXR Agonist + UDCA	OCA + UDCA	NCT05450887 (Phase III) NCT06715319 (Phase III)	Confirmation of OCA therapeutic effect	NCT05450887: Definite PBC^i^; ALP ≥1.67×ULN or TBIL >ULN but <2×ULN; On UDCA ≥6mo (stable ≥3mo) or UDCA intolerant (off UDCA ≥3mo); Age 18-75 NCT06715319: Definite PBC^i^; ALP ≥1.67×ULN or TBIL >ULN but <2×ULN; On UDCA ≥6mo (stable ≥2mo)	NCT05450887: Composite endpoint^ii^ at 12mo NCT06715319: Composite endpoint^ii^ at 6mo	NCT05450887: Completed, no results posted NCT06715319: Completed, no results posted
FXR Agonist + Fibrate	Fixed-dose Combination: OCA + Bezafibrate	NCT05239468 (Phase II) NCT06488911 (Phase III)	Dual-pathway synergistic targeted therapy	NCT05239468: Definite/probable PBC; Qualifying ALP/bilirubin; On UDCA ≥12mo or off UDCA ≥3mo NCT06488911: Completed prior studies (747-213/214) and receiving ongoing treatment	NCT05239468: Change in ALP from baseline to Week 12 NCT06488911: Number of participants with AEs/SAEs	NCT05239468: Completed, no results posted NCT06488911: Terminated, no results posted
Immunomodulator + UDCA	Methotrexate/Prednisone + UDCA	NCT00004784 (Phase III) NCT06591468 (Phase II/III)	Targeting interface hepatitis pathologic subtype	NCT00004784: PBC diagnosis^iii^; Bilirubin <3 mg/dL; ALP ≥1.5×ULN; Albumin ≥3 g/dL NCT06591468: PBC per AASLD guidelines^iv^; Moderate/severe interface hepatitis on biopsy; ALT <5×ULN; IgG <2×ULN; Anti-SMA negative; Treatment-naïve; ALP >1.67×ULN; Age 18-75	NCT00004784: Effects on pruritus, incapacitation index, serum markers; NCT06591468: Biochemical response (ALP <1.67×ULN) at 1y	NCT00004784: Completed, no results posted NCT06591468: Enrolling by invitation
Novel Agent + UDCA	Maralixibat (LUM001; Apical Sodium-Dependent Bile Acid Transporter [ASBT] Inhibitor) + UDCA	NCT01904058 (Phase II)	Exploration of bile acid reabsorption inhibition mechanism	NCT01904058: PBC diagnosis; Moderate to severe pruritus; On UDCA ≥6mo or UDCA intolerant	NCT01904058: Change from baseline in pruritus (ItchRO weekly sum score) at Week 13	NCT01904058: Completed (results: ASBT inhibitors effectively reduce bile acid pools and pruritus)
Vitamin D + UDCA	Vitamin D supplementation + UDCA	NCT06309589 (Non-drug intervention phase)	Evaluation of bone metabolism protective effects	NCT06309589: PBC diagnosis (at age ≥18); Completed ≥2y treatment with complete data; Treatment-adherent	NCT06309589: UDCA response per Paris I ^v^ and Barcelona criteria ^vi^ at 1y	NCT06309589: Completed, no results posted

This table summarizes representative studies investigating combinations where ursodeoxycholic acid (UDCA) is typically the foundational agent. Each row may include multiple trials with distinct criteria; details are provided per NCT identifier (ClinicalTrials.gov). ^i^ PBC diagnostic criteria: At least two of the following: (1) elevated ALP for ≥6 months; (2) positive AMA (immunofluorescence >1:40 or ELISA M2-positive) or PBC-specific ANA (anti-gp210/anti-sp100); (3) liver biopsy histology consistent with PBC. ^iii^ NCT00004784 diagnostic criteria: Cholestatic liver disease for ≥6 months, liver biopsy compatible with PBC, and exclusion of biliary obstruction. ^iv^ AASLD guideline diagnostic criteria: At least two of the following: (1) positive AMA or anti-gp210/anti-sp100; (2) elevated ALP; (3) liver biopsy showing non-suppurative cholangitis and destruction of interlobular bile ducts. ^v^ Paris I criteria: ALP ≤ 3 × ULN, AST ≤ 2 × ULN, and normal bilirubin; ^vi^ Barcelona criteria: ALP decrease >40% or normalization. Abbreviations: AE, adverse event; ALP, alkaline phosphatase; ALT, alanine aminotransferase; AMA, antimitochondrial antibody; ASBT, apical sodium-dependent bile acid transporter; AST, aspartate aminotransferase; BMI, body mass index; FXR, farnesoid X receptor; IgG, immunoglobulin G; LSM, liver stiffness measurement; OCA, obeticholic acid; PBC, primary biliary cholangitis; PPAR, peroxisome proliferator-activated receptor; QoL, quality of life; SAE, serious adverse event; SMA, smooth muscle antibody; TBIL, total bilirubin; TEAE, treatment-emergent adverse event; UDCA, ursodeoxycholic acid; ULN, upper limit of normal.

Metabolic Synergy: Combining UDCA with a PPAR or FXR agonist enhances correction of BA and lipid metabolism, a strategy under evaluation in multiple trials (e.g., NCT05751967, NCT05450887).

Metabolo-Immune Dual Targeting: Adding low−dose immunosuppressants (e.g., budesonide, methotrexate) to UDCA may help control adaptive immunity while addressing metabolic drivers, particularly in overlap syndromes (103).

Novel Adjunctive Targets: Emerging agents target previously inaccessible cycle components: the anti−fibrotic setanaxib (NOX1/4 inhibitor) attenuates structural reinforcement (104); senolytics clear SASP−producing senescent BECs (105); and ASBT inhibitors (e.g., odevixibat) reduce gut BA reuptake, alleviating systemic metabolic stress (106).

However, the safety of combination therapy requires careful assessment. The FXR agonist OCA can dose-dependently exacerbate pruritus and cause decreases in HDL-C and increases in LDL-C; it is contraindicated in patients with decompensated cirrhosis or signs of portal hypertension, as it may induce or worsen hepatic decompensation (93). The PPAR agonist elafibranor has a relatively neutral effect on lipid profiles (99), but fibrates should be used with caution due to myopathy risk when combined with statins (107). Combined immunosuppressants require vigilance for additive infection risk. Combination regimens should be individualized based on baseline liver function, pruritus severity, and lipid levels.

In clinical trial design, specific endpoints for measuring “cycle interruption” must be clearly defined. Current evidence supports a composite assessment of the following dimensions: (1) Biochemical response: remains the primary surrogate endpoint recognized by the US Food and Drug Administration and the European Medicines Agency, with common criteria including ALP <1.67×ULN and normal total bilirubin (93). (2) Histological fibrosis improvement: fibrosis stage is an independent prognostic predictor beyond biochemical response; liver stiffness measurement serves as a non-invasive surrogate that enhances prognostic accuracy when combined with biochemical response: liver stiffness <10 kPa carries <1% 3-year liver-related event risk, while 10–15–20 kPa stratification represents progressively increasing relative risk (108). (3) Patient-reported outcomes: pruritus and fatigue profoundly impact quality of life; both seladelpar and elafibranor improve pruritus (99). (4) Transplant-free survival as a hard endpoint: the negative results of the COBALT trial indicate that biochemical improvement alone is insufficient to infer survival benefit (93). Validation of “cycle interruption” should employ composite endpoints integrating these dimensions, with differential weighting across disease stages.

Based on the individual heterogeneity of the metabolo-immune-senescence cycle, future research should establish biomarker-driven patient stratification systems to guide combination therapy selection. Serum fibroblast growth factor 19 (FGF19) levels can reflect intestinal FXR activity, while hepatic FXR target gene (e.g., *CYP7A1*, small heterodimer partner SHP) expression profiles can assess the status of the hepatic BA synthesis feedback loop (109); in PBC patients, *CYP7A1* mRNA expression is significantly suppressed, with *FXR* and *SHP* expression also showing downward trends, suggesting adaptive regulation of BA metabolism (110). Circulating SASP-related factors (interleukin-6, CXCL10, transforming growth factor-β) as senescence burden markers have been demonstrated to correlate with disease activity in hepatic fibrosis (111). The aforementioned “biomarker-guided adaptive design” approach requires validation in prospective cohorts to establish its value in predicting treatment responses. Furthermore, exploration of novel delivery systems (such as liver-targeted nanoparticles and extracellular vesicles) and bispecific antibody technologies may provide a technical foundation for precise delivery of cycle-node-targeted therapeutics; adaptive clinical trial designs with “cycle interruption” as a composite endpoint will offer a methodological framework for validating the efficacy of such precision strategies.

## Conclusions and future perspectives

In summary, this review advances the paradigm that PBC is fundamentally a metabolo-immune disorder, whose pathogenesis is propelled by a self-sustaining “metabolo-immune-fibrosis” vicious cycle. The BEC sits at the epicenter of this cycle, where initial metabolic insults (BA toxicity, lipotoxicity) and subsequent immune attacks converge, driving a pathological process that culminates in irreversible biliary destruction.

Metabolic imbalance initiates a vicious cycle of immune response and immune backlash metabolism, which explains why the effects of single drugs do not persist and also suggests that, in the future, multiple nodes must be targeted simultaneously. By utilizing single-cell multi-omics, spatial transcriptomics, and metabolomics, patients can be categorized into different metabolo-immune subtypes. Intelligent multi-target methods such as senolytics, engineered bacteria, ASBT inhibitors, or exosome delivery can be used to accurately target each node, while dynamic markers of “circulatory interruption” can be used as experimental and clinical endpoints to evolve PBC from a “chronic autoimmune” disease to one of “functional cure”.
